# A92 EDUCATIONAL INTERVENTIONS TO IMPROVE ERGONOMIC PERFORMANCE IN GASTROINTESTINAL ENDOSCOPY: A SYSTEMATIC REVIEW

**DOI:** 10.1093/jcag/gwab049.091

**Published:** 2022-02-21

**Authors:** R Bansal, M Scaffidi, N Gimpaya, A Fecso, R Khan, J Li, N Torabi, A Shergill, S Grover

**Affiliations:** 1 St. Michael’s Hospital, Toronto, ON, Canada; 2 Department of Medicine, University of Toronto, Toronto, ON, Canada; 3 St. Michael’s Hospital, Richmond Hill, ON, Canada; 4 San Francisco VA Health Care System, San Francisco, CA

## Abstract

**Background:**

Physicians in procedural specialties, such as gastrointestinal endoscopy, are at a high risk of musculoskeletal injuries (MSPI) which can affect physician wellness and productivity. Training in ergonomic principles for endoscopy may help reduce and prevent the incidence of MSPI.

**Aims:**

The aim of this systematic review was to identify educational interventions using ergonomic strategies that target the reduction of MSPI and/or pain from GI endoscopy.

**Methods:**

We conducted a systematic search following PRESS guidelines in MEDLINE, EMBASE, PsycINFO, Web of Science, Scopus, the Cochrane Central Register of Controlled Trials and the Cochrane Database of Systematic Reviews for articles published from inception to December 16, 2020. Studies were included if they investigated educational interventions aimed at changing knowledge and/or behaviours related to ergonomics in gastrointestinal endoscopy. After screening and full-text review, we extracted data on the study design, participants, type of training and assessment of primary outcomes. We evaluated study quality with the Medical Education Research Study Quality Instrument (MERSQI). A qualitative synthesis of the data was conducted.

**Results:**

Of the initial 575 records identified in the search, 5 met inclusion criteria for qualitative synthesis. We found that most studies (n=4, 90%) were single armed interventional studies that were conducted in simulated and/or clinical settings. The most common types of intervention were didactic sessions and/or videos (n=4, 80%). Other interventions included individualized feedback (n=2, 40%), checklists (n=2, 40%), and simulated training (n=1, 20%). Two (40%) studies used both standardized assessment studies and formal statistical analyses to assess primary outcomes. All included studies reported a benefit of their interventions on their respective dimensions assessed for ergonomics. The mean MERSQI score was 9.7.

**Conclusions:**

There is emerging literature demonstrating the effectiveness of interventions to improve ergonomic performance in gastrointestinal endoscopy, which is likely to reduce MSPI among endoscopists. Further higher quality research in this field is required to make robust recommendations.

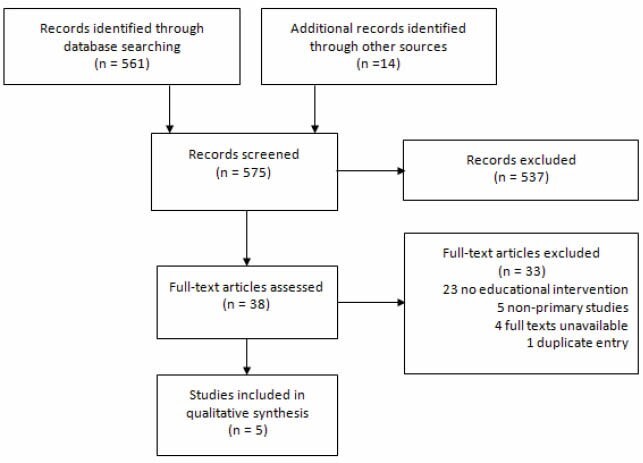

Study flow diagram

**Funding Agencies:**

None

